# Effects of HIV Infection on the Metabolic and Hormonal Status of Children with Severe Acute Malnutrition

**DOI:** 10.1371/journal.pone.0102233

**Published:** 2014-07-22

**Authors:** Aaloke Mody, Sarah Bartz, Christoph P. Hornik, Tonny Kiyimba, James Bain, Michael Muehlbauer, Elizabeth Kiboneka, Robert Stevens, John V. St. Peter, Christopher B. Newgard, John Bartlett, Michael Freemark

**Affiliations:** 1 Division of Pediatric Endocrinology and Diabetes, Pediatric Division of Quantitative Sciences, Duke University Medical Center, Durham, North Carolina, United States of America; 2 Duke Clinical Research Institute, Duke University Medical Center, Durham, North Carolina, United States of America; 3 Sarah W. Stedman Nutrition and Metabolism Center, Duke University Medical Center, Durham, North Carolina, United States of America; 4 Duke Global Health Institute, Duke University Medical Center, Durham, North Carolina, United States of America; 5 Global Research & Development, Long Term Research, PepsiCo, Inc., Purchase, New York, United States of America; 6 Mwanamugimu Nutrition Unit, Mulago Hospital Complex, Kampala, Uganda; Tulane University, United States of America

## Abstract

**Background:**

HIV infection occurs in 30% of children with severe acute malnutrition in sub-Saharan Africa. Effects of HIV on the pathophysiology and recovery from malnutrition are poorly understood.

**Methods:**

We conducted a prospective cohort study of 75 severely malnourished Ugandan children. HIV status/CD4 counts were assessed at baseline; auxologic data and blood samples were obtained at admission and after 14 days of inpatient treatment. We utilized metabolomic profiling to characterize effects of HIV infection on metabolic status and subsequent responses to nutritional therapy.

**Findings:**

At admission, patients (mean age 16.3 mo) had growth failure (mean W/H z-score −4.27 in non-edematous patients) that improved with formula feeding (mean increase 1.00). 24% (18/75) were HIV-infected. Nine children died within the first 14 days of hospitalization; mortality was higher for HIV-infected patients (33% v. 5%, OR = 8.83). HIV-infected and HIV-negative children presented with elevated NEFA, ketones, and even-numbered acylcarnitines and reductions in albumin and amino acids. Leptin, adiponectin, insulin, and IGF-1 levels were low while growth hormone, cortisol, and ghrelin levels were high. At baseline, HIV-infected patients had higher triglycerides, ketones, and even-chain acylcarnitines and lower leptin and adiponectin levels than HIV-negative patients. Leptin levels rose in all patients following nutritional intervention, but adiponectin levels remained depressed in HIV-infected children. Baseline hypoleptinemia and hypoadiponectinemia were associated with increased mortality.

**Conclusions:**

Our findings suggest a critical interplay between HIV infection and adipose tissue storage and function in the adaptation to malnutrition. Hypoleptinemia and hypoadiponectinemia may contribute to high mortality rates among malnourished, HIV-infected children.

## Introduction

Malnutrition is a major determinant of morbidity and mortality in the developing world and is the underlying cause of 3.5 million child deaths each year [1]. Poor nutrition increases greatly a child's risk of dying from diarrhea, pneumonia, measles, and malaria and is associated with decreased adult height, lower educational achievement, lower socioeconomic status, and a possible increase in chronic diseases during adulthood [2], [3]. Worldwide, malnutrition represents 35% of the burden of disease in children less than five years of age and 11% of disability-adjusted life years (DALYs) [1].

In sub-Saharan Africa, 30% of children with severe acute malnutrition (SAM) are infected with HIV, which increases mortality rates substantially; those with CD4 count <20% are at greatest risk [4], [5]. However, the factors underlying the increased risk of mortality from HIV are poorly understood. Rates of pneumonia (68%), urinary tract infection (26%), and bacteremia (18%) are comparable in severely malnourished HIV-infected and HIV-negative children [6]. Furthermore, among those who survive, the rates of nutritional recovery are similar [7]. There is consequently a critical need to elucidate the pathophysiology of SAM in children with concurrent HIV infection.

In a previous study we used metabolomic profiling to characterize changes in various hormones, growth factors, cytokines, and metabolites during nutritional rehabilitation of severely malnourished Ugandan children [8]. Here we characterized differences in baseline metabolic and hormonal status between HIV-infected and HIV-negative children with SAM and compared their subsequent responses to nutritional therapy. We hypothesized that HIV infection would modify the hormonal and metabolic responses to malnutrition and nutrient therapy and that hormones and metabolites measured at baseline might be associated with mortality in HIV-infected children.

## Methods

### Study Cohort

The study was conducted at Mwanamugimu Nutrition Unit at Mulago Hospital, in Kampala, Uganda. Children ages six months to five years who met WHO criteria for SAM were eligible for enrollment. SAM was defined as having a weight-for-height z-score (W/H z) <−3, mid-upper arm circumference (MUAC) <11.5 cm, or bilateral pitting edema. Referrals came from the Mulago pediatric acute care unit (emergency department) and community clinics in and around Kampala.

### Study Variables

A complete medical and diet history, sociodemographic profile, and physical exam including anthropometric data were obtained at time of enrollment. Lab studies included CBC and differential, blood smear, and CD4/CD8 counts (FACSCalibur, BD Biosciences, USA). HIV status was assessed using an HIV rapid antibody test (Determine, Abbott, USA; STAT-Pak, Chembio Diagnostics, USA; Uni-Gold, Trinity Biotech, Ireland) for patients >18 months of age and HIV DNA PCR (AMPLICOR HIV-1 Monitor Test version 1.5, Roche, USA) for patients <18 months. Children whose mothers had a documented negative HIV test within the previous 30 days were presumed to be HIV negative. Children with known HIV infection did not have repeat HIV testing. Those with malaria were treated with anti-malarials. All patients received counseling from a trained HIV counselor at Mwanamugimu Nutrition Unit before delivering results; HIV-infected patients were referred for appropriate HIV-related care.

### Nutritional Interventions

Nutrition rehabilitation and management of medical complications were carried out according to WHO guidelines for inpatient treatment of SAM by medical house officers at Mwanamugimu Nutrition Unit [9]. Inpatient therapy was administered in two phases according to WHO guidelines: an initial stabilization phase during which acute medical conditions were managed; and a longer rehabilitation phase once clinical status improved. Patients were fed F75 mild-based liquid formula (75 kcal and 0.9 g protein/100 mL) during the first phase and F100 (100 kcal and 2.9 g protein/100 mL) during the rehabilitation phase. Micronutrient deficiencies were corrected with vitamin A, folic acid, zinc, and iron. All patients received empiric antibiotics [9]. Patients were followed from time of enrollment until death or discharge from the inpatient unit.

### Metabolomic Analysis

Blood samples (maximum 5 mL) were collected at time of enrollment (within 24 h of admission). A second blood sample was collected after 14 days of inpatient treatment or at time of discharge from the inpatient unit, whichever occurred first. Aprotinin (500 KIU/mL of blood; Sigma-Aldrich, USA) was added to prevent protein degradation. Blood samples were collected on ice and processed promptly; EDTA plasma was stored at −70°C and shipped in bulk to the Duke University Stedman Nutrition Center for analysis. Detailed methods of the metabolic and hormonal analyses are described in the **Supporting Information (Methods S1)**.

### Statistical Analysis

Sample size was based on commonly reported concentrations and variability of classical hormones (insulin, growth hormone, cortisol) in infants and children. We evaluated pre-treatment anthropometric variables and biomarkers using non-parametric Wilcoxon Rank-Sum and absolute change during treatment using Wilcoxon Signed-Rank based on variables of interest (HIV status, mortality). The association between HIV status and mortality was assessed utilizing an odds ratio. We performed multivariable logistic and linear regression to evaluate associations between biomarkers levels, HIV status, and mortality. Analysis of leptin and total and high molecular weight (HMW) adiponectin excluded patients taking antiretroviral drugs (ARVs), given the known effects of these medications on these hormones [10]–[14]. Edematous children were excluded from analysis of anthropometric variables based on weight. All analyses were conducted using JMP Pro 9.0 (Cary, NC); a two-sided p-value<0.05 was considered statistically significant for all tests.

### Ethical considerations

The study protocol was approved by the institutional review boards at Duke University, Makerere University School of Public Health, and the Uganda National Council of Science and Technology. Sponsors of the study assisted with data interpretation but had no role in study design, data collection, or data analysis.

Written informed consent (in English and Luganda) to participate in the study and all its components was obtained from all guardians. Each patient received an insecticide-treated bed net at enrollment and transportation money to return home at the time of discharge as compensation for participation.

## Results

### Study Population

**Figure 1:**
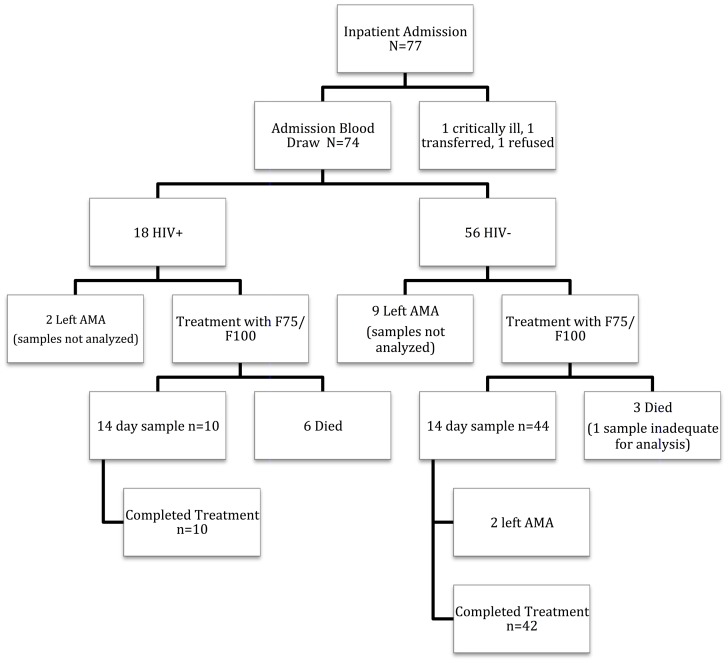
Flowchart of Patient Outcomes.

A total of 77 patients were referred to Mwanamugimu Inpatient Nutrition Unit and screened for study enrollment between December 2010 and March 2011. One patient refused to participate, another was deemed clinically unstable by the medical house officer for extra blood draws, and a third was transferred from the ward after only HIV status was assessed. Therefore, 75 patients had known HIV status and 74 had complete admission anthropometry. Analyses of hormones, metabolites, and cytokines were performed on blood samples from 62 patients at admission (16 HIV-infected including three on ARVs and 46 HIV-negative); 54 of these patients had repeat samples analyzed after 14 days of hospitalization and eight patients died before the second sample was obtained. Initial samples were insufficient in one additional patient (who died) and were not analyzed in 11 patients who left the ward prior to completing at least 14 days of treatment (including two HIV-infected patients, one of whom was on ARVs) (**Figure 1**).

The patient population was 57.3% male; mean age was 16.3±1.0 months (mean±SE). 56.8% (42/74) presented with edematous malnutrition. Non-edematous children had an initial W/H z-score −4.27±0.24 and MUAC 9.8±0.2 cm. Mean length-for-age (L/A) z-score was −2.97±0.18; head circumference-for-age (HC/A) z-score was −1.15±0.18. 9.5% (7/74) had malaria; one HIV-infected patient had concurrent malaria (**Table 1**).

**Table 1:** Baseline Anthropometric and Hematologic Characteristics of HIV-infected and HIV-negative Patients.

	HIV-infected (n = 18)	HIV-negative (n = 56)	
	Number (%)		p-value
Male Sex	10/18 (55.6)	32/56 (57.1)	1.00
Edema Present	9/18 (50)	33/56 (58.9)	0.589
Positive Malaria Smear	1/18 (5.6)	6/56 (10.7))	0.555
Newly Diagnosed HIV infection	12/18 (66.7)	-	-
Current ARV treatment	4/6 (66.7)	-	-
Mortality	6/18 (33.3)	3/56 (5.4)	**0.0051**
	**Mean±SEM**		
Age	19.2±2.6	15.4±1.1	0.315
Days in Treatment	26.4±3.9	24.9±1.5	0.850
**Admission Anthropometry**			
W/H % (nonedematous)	70.5±2.4	71.3±1.4	0.571
W/H Z-Score (nonedematous)	−4.44±0.48	−4.20±0.28	0.615
W/A Z-Score (nonedematous)	−5.14±0.61	−4.87±0.33	0.870
MUAC (nonedematous)	9.6±0.5	9.9±0.2	0.599
L/A Z-score (all patients)	−3.17±0.40	−2.90±0.20	0.492
H/C Z-score (all patients)	−0.72±0.32	−1.25±0.21	0.246
**Hematology**			
Abs CD4 Count	644±103	2734±253	**<0.0001**
CD4%	14.6±2.1	34.8±1.2	**<0.0001**
Abs CD8 Count	1724±387	1610±120	0.638
CD8%	37.3±2.6	21.3±1.0	**<0.0001**
CD4/CD8 Ratio	0.44±0.08	1.83±0.10	**<0.0001**
WBC (10^3^/µl)	8.5±1.2	13.2±1.0	**0.0082**
Hemoglobin (g/dL)	7.7±0.4	9.0±0.2	**0.0130**
Platelets (10^3^/µl)	267±37	371±24	**0.0231**

As previously reported, overall mortality was 12.2% (9/74). Of those who successfully completed inpatient nutritional rehabilitation, mean length of stay was 25.2 days. During hospitalization, mean W/H z-score increased 1.00±0.18 in non-edematous children. Among surviving patients, 80% (52/65) were followed until discharge and 20% (13/65) left the ward against medical advice before achieving nutritional stability.

### Baseline Characteristics of HIV-infected and HIV-negative Patients

HIV prevalence in the study population was 24% (18/75); two-thirds (12/18) of these were newly diagnosed HIV infections. Four of the six previously diagnosed patients were being treated with ARVs upon admission (**Table 1**).

Similar proportions of HIV-infected and HIV-negative patients presented with edematous malnutrition (p = 0.589). Non-edematous HIV-infected and HIV-negative patients presented with similar degrees of wasting (W/H z-score −4.44 vs. −4.20, p = 0.615). HIV-infected children had lower absolute CD4 counts (644 vs. 2734, p<0.0001), WBC counts, hemoglobin, and platelets (**Table 1**).

HIV-infected patients had increased mortality rates: 33.3% for seropositive children compared with 5.4% for seronegative children (OR = 8.83, CI 1.93–40.43, p = 0.0051). Among those who survived, there were similar improvements in W/H z in (non-edematous) HIV-infected and HIV-negative patients after 14 days (0.85 vs. 0.43, p = 0.412).

### Effects of HIV Infection on Baseline Metabolic Profile

Both HIV-infected and HIV-negative patients presented in a severe catabolic state with exaggerated lipolysis, fatty acid oxidation, and hypoaminoacidemia (**Table 2** **and** **3**). Non-esterified fatty acids (NEFA), ketones, C2 acyl (acetyl)carnitine, and even-chained acylcarnitine molar sum were elevated in both groups at presentation, though ketones (p = 0.039), acetylcarnitine (p = 0.0103), and even-chained acylcarnitine molar sum (p = 0.0108) were higher in HIV-infected patients. Levels of albumin, amino acids, and C3 acyl (propionyl)carnitine, a byproduct of branched chain amino acid catabolism, were comparably low in both groups. Yet blood glucose levels were maintained in the normal range. Triglycerides were higher in HIV-infected patients (p<0.001). Moreover, a number of inflammatory markers, including CRP, IL-2, IL-6, IL-8, and TNF-α were higher in HIV-infected patients, with IL-2 (p = 0.016) and TNF-α (p = 0.025) reaching statistical significance (**Table 2**). Edematous patients had higher alanine amino transferase (ALT) and gamma glutamyl transpeptidase (GGT) levels and lower albumin and amino acid levels than non-edematous patients (data not shown); these metrics did not differ among the HIV-infected and HIV-negative groups. Edematous patients also had lower total and HMW adiponectin levels; however, edema did not modify the association between HIV status and hypoadiponectinemia.

**Table 2:** Baseline Metabolic Profile of HIV-infected and HIV-negative Patients.

	HIV-infected (n = 16)	HIV-negative (n = 46)	
	Mean±SEM		p-value
**Fatty Acid Metabolites**			
NEFA (mmol/L)	0.65±0.10	0.54±0.06	0.285
Total Ketones (µmol/L)	826±259	424±95	**0.0387**
**Acylcarnitines**			
C2 (µmol/L)	22.3±3.5	14.4±2.4	0.0103
C3 (µmol/L)	0.47±0.07	0.38±0.04	0.179
C2/C3 Ratio	54.3±9.7	44.7±5.7	0.195
Even-Chain Acylcarnitine Molar Sum (µmol/L)	24.0±3.7	16.0±2.6	**0.0108**
**Hormones**			
Insulin (µIU/ml)	1.81±0.48	2.45±0.45	0.321
Growth Hormone (ng/ml)	12.4±2.7	11.0±1.3	0.380
IGF-1 (ng/ml)	13.3±4.4	9.4±1.7	0.597
Total Ghrelin (pg/ml)	3577±566	4040±320	0.435
GLP-1 (pg/ml)	128.8±21.3	96.0±12.8	0.106
PYY (pg/ml)	1195±160	1202±105	0.866
Cortisol (µg/dl)	54.0±3.2	46.0±2.7	0.106
**Adipocytokines** *	**n = 13**	**n = 46**	
Leptin (pg/ml)	69.8±26.6	292±52	**0.0163**
Total Adiponectin (ng/ml)	8049±1081	15268±1133	**0.0017**
HMW Adiponectin (ng/ml)	4409±757	9356±761	**0.0014**
**Amino Acids**			
Amino Acid Molar Sum (µmol/L)	1230±62	1190±51	0.417
**Inflammatory Cytokines**			
IL-2 (pg/ml)	7.7±2.7	3.6±1.2	**0.0158**
IL-6 (pg/ml)	96.3±58.4	25.6±15.9	0.139
IL-8 (pg/ml)	299.3±191.0	75.2±25.9	0.060
TNF-α (pg/ml)	43.0±5.5	37.4±9.5	**0.0248**
**Other**			
Glucose (mg/dl)	77.1±7.9	85.9±3.9	0.474
Creatinine (mg/dl)	0.30±0.04	0.27±0.03	0.296
Phosphorus (mg/dl)	2.99±1.40	3.28±1.02	0.390
Albumin (g/dl)	2.0±0.2	2.0±0.1	0.847
CRP (mg/L)	63.7±16.5	26.8±5.3	0.0730
Triglycerides (mg/dl)	177.6±14.0	122.9±12.2	**0.0008**

*Excludes patients on ARVs.

**Table 3:** Baseline Amino Acid Levels of HIV-infected and HIV-negative patients.

	HIV-infected (n = 16)	HIV-negative (n = 46)	
	Mean±SEM		p-value
Glycine (µmol/L)	235±21.4	237±12.6	0.866
Alanine	153±27.4	217±16.2	**0.0330**
Serine	99.2±99.5	113±5.6	0.464
Proline	152±14.4	153±8.5	0.904
Valine	100±10.7	75.6±6.3	**0.0248**
Leucine/Isoleucine	82.0±8.9	70.5±5.2	0.237
Methionine	16.1±1.7	15.6±1.0	0.742
Histidine	69.1±7.0	53.3±4.1	0.250
Phenylalanine	79.6±7.7	43.0±4.5	**0.0067**
Tyrosine	30.1±4.6	22.6±2.7	0.207
Aspartate	39.2±4.8	36.5±2.8	0.853
Glutamate	111±10.5	91.3±6.2	0.435
Ornithine	27.4±3.5	26.2±2.1	0.381
Citrulline	8.9±1.2	8.2±0.7	0.421
Arginine	28.3±3.3	27.7±1.9	0.323

Insulin and IGF-1 levels were low in both HIV-infected and HIV-negative subjects, while growth hormone (GH), ghrelin, cortisol, GLP-1, and peptide YY (PYY) were high (compare levels to those in references [15]–[18]). Excluding analysis of three patients taking ARVs, which are known to affect adipose tissue function, the levels of leptin (p = 0.016), total adiponectin (p = 0.0017), and high molecular weight (HMW) adiponectin (p = 0.0014) were significantly lower in HIV-infected than in HIV-negative subjects (**Table 2**). Multivariate logistic regression controlling for the degree of wasting (as assessed by W/H z-score) established that HIV infection was associated with lower total adiponectin (p = 0.0113) and HMW adiponectin (p = 0.009), but not lower leptin (p = 0.157) (**Table 4**).

**Table 4:** Multivariate regression assessing the effect of HIV status on leptin, total adiponectin, and HMW adiponectin when controlling for admission W/H z-score.

	Beta (HIV status)	p-value	Adjusted R^2^
Leptin	63.7±44.4	0.1573	0.294
Total Adiponectin	2752±1051	**0.0113**	0.274
HMW Adiponectin	1949±720	**0.0090**	0.253

### Effects of HIV Infection on Metabolic Response to Nutritional Rehabilitation

54 patients had blood samples drawn both at admission and after 14 days of treatment. We were unable to obtain day-14 samples in any of the patients who died.

**Figure 2:**
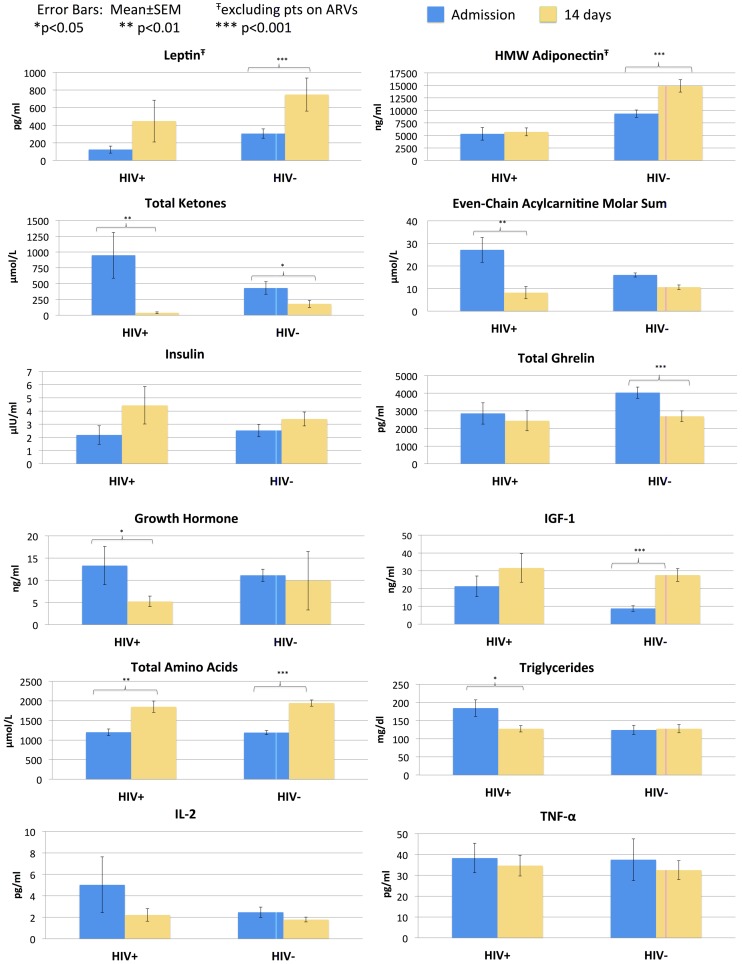
Comparison of metabolic response to inpatient rehabilitation in 54 patients who completed treatment (10 HIV-infected and 44 HIV-negative children). Analysis of leptin and HMW adiponectin excluded those patients taking ARVs. Data are represented as the mean±SEM.

HIV-infected and HIV-negative patients demonstrated similar trends in most metabolites, hormones, and cytokines in response to nutritional treatment. NEFA, total ketones, and even-chained acylcarnitines decreased (**Figure 2** **and** **Table 5**), while albumin and the majority of amino acids increased (**Table 6**). There was a rise in the levels of propionylcarnitine (HIV-infected: 0.50 vs. 0.65, p = 0.2754; HIV-negative 0.36 vs. 0.67, p<0.0001), likely reflecting the catabolism of (newly available) dietary branched chain amino acids [19]. Plasma insulin, IGF-1, and leptin increased while plasma ghrelin, GH, and cortisol declined. The majority of inflammatory markers decreased in both groups (statistically significant for IL-6 and CRP). After 14 days of nutritional therapy, there were no significant differences among surviving HIV-infected and HIV-negative patients with respect to NEFA, total ketones, insulin, IGF-1, leptin, ghrelin, GH, cortisol, GLP-1, PYY, IL2, IL6, IL8, or TNF-α levels (**Figure 2** **and** **Table 5**).

**Table 5:** Changes in Metabolic Profiles of HIV-infected (surviving) and HIV-negative patients.

	HIV-infected (n = 10)			HIV-negative (n = 44)		
	Mean±SEM		p-value	Mean±SEM		p-value
	Admission	14-day		Admission	14-day	
**Fatty Acid Metabolites**						
NEFA (mmol/L)	0.72±0.16	0.24±0.06	**0.0020**	0.53±0.06	0.36±0.037	0.0723
Total Ketones (µmol/L)	948±362	39±14	**0.0098**	431±99	179±56	**0.0256**
**Acylcarnitines**						
C2 (µmol/L)	25.2±5.2	7.2±0.8	**0.0098**	14.5±2.6	9.3±0.94	0.171
C3 (µmol/L)	0.50±0.11	0.65±0.11	0.275	0.36±0.03	0.67±0.06	**<0.0001**
C2/C3 Ratio	60.6±14.7	13.4±2.0	**0.0020**	45.7±5.9	22.5±4.5	**<0.0001**
Even-Chain Acylcarnitine Molar Sum (µmol/L)	27.1±5.5	8.2±0.9	**0.0098**	16±2.7	10.6±1	0.216
**Hormones**						
Insulin (µIU/ml)	2.18±0.70	4.43±1.42	0.084	2.52±0.47	3.4±0.53	0.108
Growth Hormone (ng/ml	13.3±4.3	5.2±1.2	**0.027**	11.2±1.4	10.1±1.7	0.147
IGF-1 (ng/ml)	21.3±5.8	31.6±8.1	0.250	8.8±1.6	27.6±3.6	**<0.0001**
Total Ghrelin (pg/ml)	2851±603	2439±570	0.084	4029±319	2692±303	**<0.0001**
GLP-1 (pg/ml)	116.7±27.5	86.0±22.1	0.375	93.6±13.0	88.8±12.4	0.863
PYY (pg/ml)	916±118	893±112	1.0	1144±101	1011±78	0.100
Cortisol (µg/dl)	56.6±3.4	41.1±6.7	0.0625	45.3±2.8	38.3±3	**0.0123**
**Adipocytokines** *	**n = 7**			**n = 44**		
Leptin (pg/ml)	123.1±39.7	446±238	0.128	305±53.5	748.8±188	**0.0011**
Total Adiponectin (ng/ml)	8383±1569	10154±1815	0.297	15115±1093	20792±1329	**<0.0001**
HMW Adiponectin (ng/ml)	5308±1259	5717±795	0.625	9350±748	14895±1245	**<0.0001**
**Amino Acids**						
Amino Acid Molar Sum (µmol/L)	1198±84.4	1850±145	**0.005**	1192±53.6	1944±80.5	**<0.0001**
**Inflammatory Cytokines**						
IL-2 (pg/ml)	5.0±2.6	2.2±0.6	0.477	2.5±0.5	1.8±0.2	0.255
IL-6 (pg/ml)	49±32	3.4±0.7	**0.0371**	9.9±1.5	7.8±2.8	**0.0256**
IL-8 (pg/ml)	344±300	29.7±3.4	0.0645	50.6±9.0	41.7±4.4	0.638
TNF-α (pg/ml)	38.3±7.1	34.7±5	0.922	37.5±10	32.5±4.5	1.0
**Other**						
Glucose (mg/dl)	72.1±9.3	75.4±3.7	0.625	84.2±3.8	77.0±2.2	0.0634
Creatinine (mg/dl)	0.32±0.06	0.36±0.11	0.910	0.25±0.03	0.32±0.07	0.694
Phosphorus (mg/dl)	3.2±0.4	4.2±0.2	0.086	3.4±0.2	4.6±0.1	**<0.0001**
Albumin (g/dl)	2.03±0.32	2.28±0.20	0.219	2.01±0.17	2.51±0.12	**<0.0001**
CRP (mg/L)	70.7±21.1	14.2±7.2	**0.0391**	26.8±5.7	9.9±4.1	**0.0003**
Triglycerides (mg/dl)	184±22.8	127±8.7	**0.0391**	124.3±12.7	127.7±11.2	0.416

*Excludes patients on ARVs.

**Table 6:** Changes in Amino Acid Levels of HIV-infected (surviving) and HIV-negative patients.

	HIV-infected (n = 10)			HIV-negative (n = 44)		
	Mean±SEM		p-value	Mean±SEM		p-value
	Admission	14-day		Admission	14-day	
Glycine (µmol/L)	216±16.6	313±28.1	**0.0020**	238±13.7	305±13.4	**<0.0001**
Alanine	150±18.1	409±57.7	**0.0020**	218±18.6	418±27.5	**<0.0001**
Serine	98.8±4.5	125±14.6	**0.0371**	113±6.4	157±6.9	**<0.0001**
Proline	155±14.5	296±42.6	**0.0098**	152±9.4	282±20.3	**<0.0001**
Valine	102±15.8	130±21.3	0.375	76.8±6.2	152±10.2	**<0.0001**
Leucine/Isoleucine	80.1±15.0	112±15.2	0.106	71.0±5.2	132±7.3	**<0.0001**
Methionine	16.8±2.8	19.7±3.3	0.193	15.6±1.0	24.5±1.6	**<0.0001**
Histidine	64.5±12.7	53.9±5.7	0.432	52.8±3.4	50.7±2.7	0.954
Phenylalanine	78.4±18.2	59.8±5.3	0.432	43.3±2.8	53.0±2.5	**0.0184**
Tyrosine	29.8±7.2	45.8±10.6	0.0840	22.6±2.7	52.1±5.0	**<0.0001**
Aspartate	31.3±6.3	50.4±10.5	**0.0039**	35.4±2.7	51.0±5.0	**<0.0001**
Glutamate	109±12.7	135±17.3	0.275	91.3±4.7	152±8.2	**<0.0001**
Ornithine	29.3±3.1	40.2±6.8	**0.0195**	26.7±2.3	47.9±3.3	**<0.0001**
Citrulline	8.9±1.1	16.3±3.4	**0.0098**	8.3±0.7	19.0±1.6	**<0.0001**
Arginine	29.2±2.4	44.5±6.9	**0.0137**	27.8±2.2	49.7±3.3	**<0.0001**

However, HIV status was associated with differential effects on adiponectin levels during nutritional recovery. Excluding patients on ARVs, HIV-infected patients had no significant changes in total (8383 vs. 10154, p = 0.2969) or HMW adiponectin (5308 vs. 5717, p = 0.625). In contrast, HIV-negative patients had marked increases in total (15115 vs. 20792, p<0.0001) and HMW adiponectin (9350 vs. 14895, p<0.0001) during recovery. Furthermore, after 14 days of treatment, HIV-infected patients still had significantly lower levels of total (p = 0.0023) and HMW adiponectin (p = 0.0019) than HIV-negative patients (**Figure 2** **and** **Table 5**).

### Predictors of Mortality During Inpatient Treatment

Non-edematous patients who died had more striking manifestations of wasting than those who survived, as reflected in lower W/H z-score (−6.28 vs. −3.98, p = 0.0244) and MUAC (7.8 vs. 10.1, p = 0.0019). In all patients, there was a greater degree of stunting in those who died (L/A z-score −4.03 vs. −2.82, p = 0.0454) (**Table 7**).

**Table 7:** Baseline Characteristics and Metabolic Profiles associated with Mortality.

	Died (n = 8)	Survived (n = 54)	
	Mean±SEM		p-value
**Anthropometry (nonedematous)**			
Admission W/H %	61.1±4.6	72.5±1.0	**0.0121**
Admission W/H Z-Score	−6.28±1.05	−3.98±0.19	**0.0244**
Admission W/A Z-Score	−7.01±0.86	−4.63±0.26	**0.0221**
Admission MUAC	7.8±0.3	10.1±0.2	**0.0019**
L/A z-score (all patients)	−4.03±0.58	−2.82±0.18	**0.0454**
**Fatty Acid Metabolites**			
NEFA (mmol/L)	0.57±0.15	0.56±0.06	0.557
Total Ketones (µmol/L)	539±276	526±106	0.456
**Acylcarnitines**			
C2 (µmol/L)	16.3±2.2	16.5±2.3	0.139
C3 (µmol/L)	0.51±0.09	0.38±0.03	0.128
C2/C3 Ratio	38.5±7.7	48.4±5.5	0.858
Even-Chain Acylcarnitine Molar Sum (µmol/L)	17.9±2.2	18.1±2.5	0.139
**Hormones**			
Insulin (µIU/ml)	1.10±0.97	2.46±0.38	0.182
Growth Hormone (ng/ml)	10.5±3.4	11.5±1.3	0.442
IGF-1 (ng/ml)	5.6±4.8	11.1±1.8	0.118
Total Ghrelin (pg/ml)	4660±771	3811±297	0.361
GLP-1 (pg/ml)	149.1±30.3	97.9±11.7	0.080
PYY (pg/ml)	1866±227	1101±87	**0.0089**
Cortisol (µg/dl)	52.2±6.2	47.4±2.4	0.484
**Adipocytokines** *	**n = 8**	**n = 51**	
Leptin (pg/ml)	7.1±4.4	280±47	**0.0002**
Total Adiponectin (ng/ml)	10403±3360	14191±1017	0.075
HMW Adiponectin (ng/ml)	4894±1922	8795±693	**0.0184**
**Amino Acids**			
Amino Acid Molar Sum (µmol/L)	1248±70	1193±46	0.319
**Inflammatory Cytokines**			
IL-2 (pg/ml)	16.2±2.8	2.9±1.1	**0.0004**
IL-6 (pg/ml)	223.6±47.9	17.2±18.4	0.166
IL-8 (pg/ml)	323±147	105±57	**0.0042**
TNF-α (pg/ml)	46.8±20.2	37.6±7.8	**0.0203**

*Excludes patients on ARVs.

In addition to HIV infection, factors at baseline associated with subsequent mortality were hypoleptinemia (p = 0.0002), low levels of HMW adiponectin (p = 0.0149), and high levels of PYY (p = 0.0087), IL2 (p = 0.0004), IL6 (p = 0.004), and TNF-α (p = 0.0203) (**Table 7**) [8]. Multivariate logistic regression analysis controlling for HIV status and admission W/H z showed that hypoleptinemia at baseline remained a significant predictor of mortality (OR 0.906, CI 0.827–0.993, p = 0.035) while HMW adiponectin at baseline became insignificant. Mortality did not vary with other baseline measures including presence of edema, hemoglobin, glucose, creatinine, albumin, phosphorus, other hormones and growth factors, fatty acid metabolites, or amino acid or cytokine levels.

## Discussion

Malnutrition remains a major cause of morbidity and mortality, with the greatest impact in low-income countries. HIV infection is detected in 30% of children with SAM and is associated with greatly increased mortality rates [4]. The role of HIV in the pathophysiology of malnutrition is poorly understood. Here we characterized differences in baseline metabolic and hormonal status between HIV-infected and HIV-negative children with SAM and compared their subsequent responses to current WHO recommended nutritional therapy. A major finding of this study is that HIV-infected children with SAM present with significant reductions in the adipocytokines leptin and adiponectin that are associated with mortality during inpatient hospitalization.

In our study the prevalence of HIV infection was 24%, with two-thirds of these representing new diagnoses. Mortality in HIV-infected children was very high (33.3%), similar to results from a previous meta-analysis [4]. HIV-infected and HIV-negative patients presented with similar degrees of wasting and edema, and among those who survived, achieved similar rates of growth and recovery. A previous study found HIV-infected patients to be more wasted at baseline; nevertheless that study, like ours, noted that seropositive and seronegative patients achieve similar rates of catch-up growth during nutritional treatment and that increased wasting at presentation is associated with mortality [7].

Both HIV-infected and HIV-negative children presented in a severe catabolic state characterized by elevated NEFA, total ketones, and even-numbered acylcarnitines (derived from fatty acid oxidation) and striking reductions in serum albumin and amino acids. At the same time, blood glucose levels were maintained in the normal range. Leptin, adiponectin, insulin, and IGF-1 levels were low while growth hormone, cortisol, and ghrelin levels were high. [8] This profile suggests a state in which fat catabolism and glucose production are prioritized above energy storage and growth [8], [20]–[25]. At baseline, serum triglycerides, ketones, and even-chain acylcarnitines were higher and leptin and HMW adiponectin lower in HIV-infected patients than in HIV-negative patients. When controlling for W/H z-score, lower HMW adiponectin levels remained significantly associated with HIV infection, though leptin levels did not.

Nutritional treatment reversed the state of lipid mobilization and fatty acid oxidation and increased the levels of amino acids and C3 acyl (propionyl)carnitine. Insulin and IGF-1 rose while GH, cortisol, and ghrelin declined. HIV status did not modify the effect of treatment on most metabolites, hormones, growth factors, and cytokines. However, nutritional intervention increased HMW and total adiponectin levels in HIV-negative patients but not in HIV-infected patients; their levels remained significantly lower despite high calorie feeds. Leptin, on the other hand, increased in both HIV-infected and HIV-negative subjects.

Previous studies have linked decreased levels of leptin and adiponectin to HIV infection, particularly in the context of HIV-associated lipodystrophy, a syndrome characterized by fat redistribution, dyslipidemia, and metabolic syndrome. Many investigations implicate ARVs in the development of this syndrome, citing medication-induced adipose dysregulation and mitochondrial toxicity as potential mechanisms [10]–[14]. Additional studies, however, in untreated adults and mice have shown that HIV infection itself may be associated with adipose tissue dysfunction and decreased levels of adiponectin and leptin [26]–[28].

Adiponectin is produced by mature adipocytes; over-expression of adiponectin increases hepatic insulin sensitivity, while low levels of adiponectin are associated with insulin resistance and the metabolic syndrome [29]. Circulating leptin levels rise in proportion to white adipose tissue mass; higher levels are associated with obesity and lower levels with fasting and malnutrition [22]. The severe hypoadiponectinemia and hypoleptinemia in our HIV-infected children suggest a state of insulin resistance associated with depletion of white adipose tissue reserves.

The pre-existing mass and function of white adipose tissue appear to play roles in the adaptation to, and recovery from, malnutrition because low levels of leptin and adiponectin at baseline were associated with subsequent mortality. Indeed, baseline hypoleptinemia remained a strong predictor of mortality when controlling for HIV infection and W/H z-score in a multivariate analysis. While these findings do not prove that mortality is caused by hypoadiponectinemia and/or hypoleptinemia, there are potential mechanisms by which hypoadiponectinemia and hypoleptinemia might contribute to mortality risk. For example, a lack of pre-existing adipose tissue stores, suggested by hypoleptinemia at presentation, may limit a child's ability to sustain energy production for critical cardiorespiratory function during the initial phases of acute severe malnutrition. Moreover, leptin and adiponectin have immunoregulatory properties that may modulate the response to infectious pathogens. Leptin activates NK cells, induces neutrophil chemotaxis, enhances secretion of pro-inflammatory cytokines, and induces activation and proliferation of T-cells, while adiponectin promotes production of numerous anti-inflammatory cytokines [30], [31]. It is possible that the combined effects of HIV infection and malnutrition on adipose tissue and immune function may increase mortality risk.

There were several limitations to our study. Blood samples were not obtained after fasting, as this could not be justified in critically ill patients. Our small sample size prevented us from conducting potentially important analyses of subgroups including HIV-infected patients who died (n = 6) and those already taking ARVs. Additionally, 17.6% (n = 13) of the original patient population left the ward prior to completing nutritional rehabilitation.

Nevertheless, this study provides a comprehensive analysis of the effects of HIV on the pathophysiology and recovery from SAM in childhood. Our findings suggest a critical interplay between HIV infection and adipose tissue storage and function in the adaptation to malnutrition.

Mortality in malnutrition is predicted by low W/H z and low MUAC. However, these can be difficult or impossible to interpret in infants and children with nutritional edema. Currently, all patients with nutritional edema are categorized as having SAM and treated accordingly. Our finding that hypoleptinemia predicts mortality in edematous as well as non-edematous subjects suggests that leptin assays might in the future be used to identify and target malnourished children at highest risk of death [8].

Finally, it should be noted that the optimal timing for initiating ARV treatment in HIV-infected children with SAM is currently unknown. In some cases, early initiation of therapy may increase the risk of clinical deterioration; in clinically stable children, however, it appears to improve outcomes [12], [32]–[35]. Future studies should determine if the effects of ARVs on adipose tissue metabolism and function influence the clinical response to treatment of severely malnourished children.

## Supporting Information

Methods S1 **Detailed methods on the assays used for the metabolic and hormonal analyses.**
(DOC)
