# Supplementary material for: Effects of HIV Infection on the Metabolic and Hormonal Status of Children with Severe Acute Malnutrition
Source: PLoS One. 2014 Jul 22;9(7):e102233. doi: 10.1371/journal.pone.0102233 (PMC4106836; doi:10.1371/journal.pone.0102233)
Supplement: Methods S1 — Detailed methods on the assays used for the metabolic and hormonal analyses. (DOC) [file pone.0102233.s001.doc]

**SUPPORTING INFORMATION**

**Title: Effects of HIV infection on Metabolic and Hormonal Status in Children with Severe Acute Malnutrition**

**Methods for Metabolomic and Hormonal Analysis**

Immunoassays**.** Measurements of glucose, nonesterified fatty acids (NEFA), total ketones, lactate, albumin, C-reactive protein (CRP), triglycerides, and aspartate aminotransferase (AST) were performed using a Beckman-Coulter DxC600 clinical analyzer (Brea, CA). Reagents were provided by Beckman, except for NEFA and total ketones, which were from Wako USA (Richmond, VA). Immunoassays using electrochemiluminescent technology were performed on an SI-2400 imager from Meso Scale Discovery (MSD, Gaithersburg, MD). Plates and reagents, also from MSD, were in both singleplex format for total glucagon-like peptide-1 (GLP-1), and multiplex for cytokines (IL-1β, IL-2, IL-6, IL-8, IL-10, IL-12p70, GM-CSF, TNF-α, and IFN-γ) and insulin/leptin. Other enzyme-linked immunoassays were performed using a SpectraMax M2e spectrophotometric plate reader from Molecular Devices (Sunnyvale, CA). Assay kits for the measurement of cortisol, insulin-like growth factor-1 (IGF-1), and multimeric adiponectin (both total and high molecular weight isoforms) were provided by Alpco (Salem, NH). Those for total ghrelin and Peptide YY were from Millipore (Billerica, MA). An additional duplex assay for monocyte chemotactic protein-1 (MCP-1) and plasminogen activator inhibitor-1 (PAI-1) was performed using a Searchlight imager and kits from Aushon (Billerica, MA).

Targeted Amino Acid/Acylcarnitine Analysis. Amino acids and acylcarnitines were analyzed by flow injection, electrospray-ionization, tandem mass spectrometry and quantified by isotope dilution using previously described methods [1,2]. Briefly, plasma samples were spiked with a cocktail of heavy-isotope internal standards (Cambridge Isotope Laboratories, MA, USA; C-D-N, Isotopes, Canada) and deproteinated with methanol. The methanol supernatants were dried and esterified with either acidified methanol or butanol for acylcarnitine or amino acid analysis, respectively. Mass spectra for acylcarnitine and amino acid esters were obtained using precursor ion and neutral loss scanning methods, respectively. Spectra were acquired in a multi-channel analyzer (MCA) mode to improve signal-to-noise. Data were acquired using a Waters Acquity^TM^  UPLC system equipped with a TQ (triple quadrupole) detector and a data system controlled by MassLynx 4.1 operating system (Waters, Milford, MA). Ion ratios of analyte to respective internal standard computed from centroided spectra were converted to concentrations using calibrators constructed from authentic aliphatic acylcarnitines and amino acids (Sigma, MO, USA; Larodan Sweden) and bovine adult serum (Sigma, MO, USA). Leu/Ile are reported as a single analyte because they are not resolved by this MS/MS method. It should also be noted that the Leu/Ile values include contributions from allo-isoleucine and hydroxyproline. Under normal circumstances these isobaric amino acids contribute little to the signal attributed to Leu/Ile (f)**.** In addition, the acidic conditions used to form butyl esters results in partial hydrolysis of glutamine to glutamic acid and of asparagine to aspartate. Accordingly, values reported as Glu/Gln or Asp/Asn are not meant to signify the molar sum of glutamate and glutamine, or of aspartate and asparagine, but rather measure the amount of glutamate or aspartate plus the contribution of the partial hydrolysis reactions of glutamine and asparagine.

**REFERENCES**

1. An J, Muoio DM, Shiota M, Fujimoto Y, Cline GW, et al. (2004) Hepatic expression of malonyl-CoA decarboxylase reverses muscle, liver and whole-animal insulin resistance. Nat Med 10: 268-274.

2. Newgard CB, An J, Bain JR, Muehlbauer MJ, Stevens RD, et al. (2009) A branched-chain amino acid-related metabolic signature that differentiates obese and lean humans and contributes to insulin resistance. Cell Metab 9: 311-326.
